# Syncytia Formation in Oncolytic Virotherapy

**DOI:** 10.1016/j.omto.2019.09.006

**Published:** 2019-10-01

**Authors:** Chase Burton, Eric Bartee

**Affiliations:** 1 Department of Microbiology and Immunology, Medical University of South Carolina, Charleston, SC 29425, USA

**Keywords:** syncytia, oncolytic virotherapy, fusogenic virus, fusion protein

## Abstract

Oncolytic virotherapy uses replication-competent virus as a means of treating cancer. Whereas this field has shown great promise as a viable treatment method, the limited spread of these viruses throughout the tumor microenvironment remains a major challenge. To overcome this issue, researchers have begun looking at syncytia formation as a novel method of increasing viral spread. Several naturally occurring fusogenic viruses have been shown to possess strong oncolytic potential and have since been studied to gain insight into how this process benefits oncolytic virotherapy. Whereas these naturally fusogenic viruses have been beneficial, there are still challenges associated with their regular use. Because of this, engineered/recombinant fusogenic viruses have also been created that enhance nonfusogenic oncolytic viruses with the beneficial property of syncytia formation. The purpose of this review is to examine the existing body of literature on syncytia formation in oncolytics and offer direction for potential future studies.

## Main Text

Roughly 1.6 million new cases of cancer are diagnosed each year, leading to an ever-increasing number of patients in need of viable treatment options.^1^ Whereas there have been many advances in cancer treatment, most patients ultimately still undergo chemotherapy and/or radiation therapy as their standard of care. Unfortunately, these treatments are associated with varying amounts of success, and many patients experience either refractory or relapsing disease. Due to these underwhelming results, medicine has long been searching for more efficient solutions to the problem of cancer.

One solution that has recently shown great promise is the field of oncolytic virotherapy (OV).^2^ This treatment uses cancer-tropic viruses to infect and subsequently eliminate a wide range of malignancies. The power of the oncolytic strategy is 2-fold. First, it combines a multimodal therapeutic approach, which is both rapid, through the direct cellular death caused from viral infections, as well as long term, through the initiation of an adaptive immune response against both viral and tumor antigens.^3^, ^4^, ^5^, ^6^ Second, genetic engineering allows for “arming” of the oncolytic genomes to maximize phenotypes, which are associated with improved treatment efficacy.^7^

**Figure 1:**
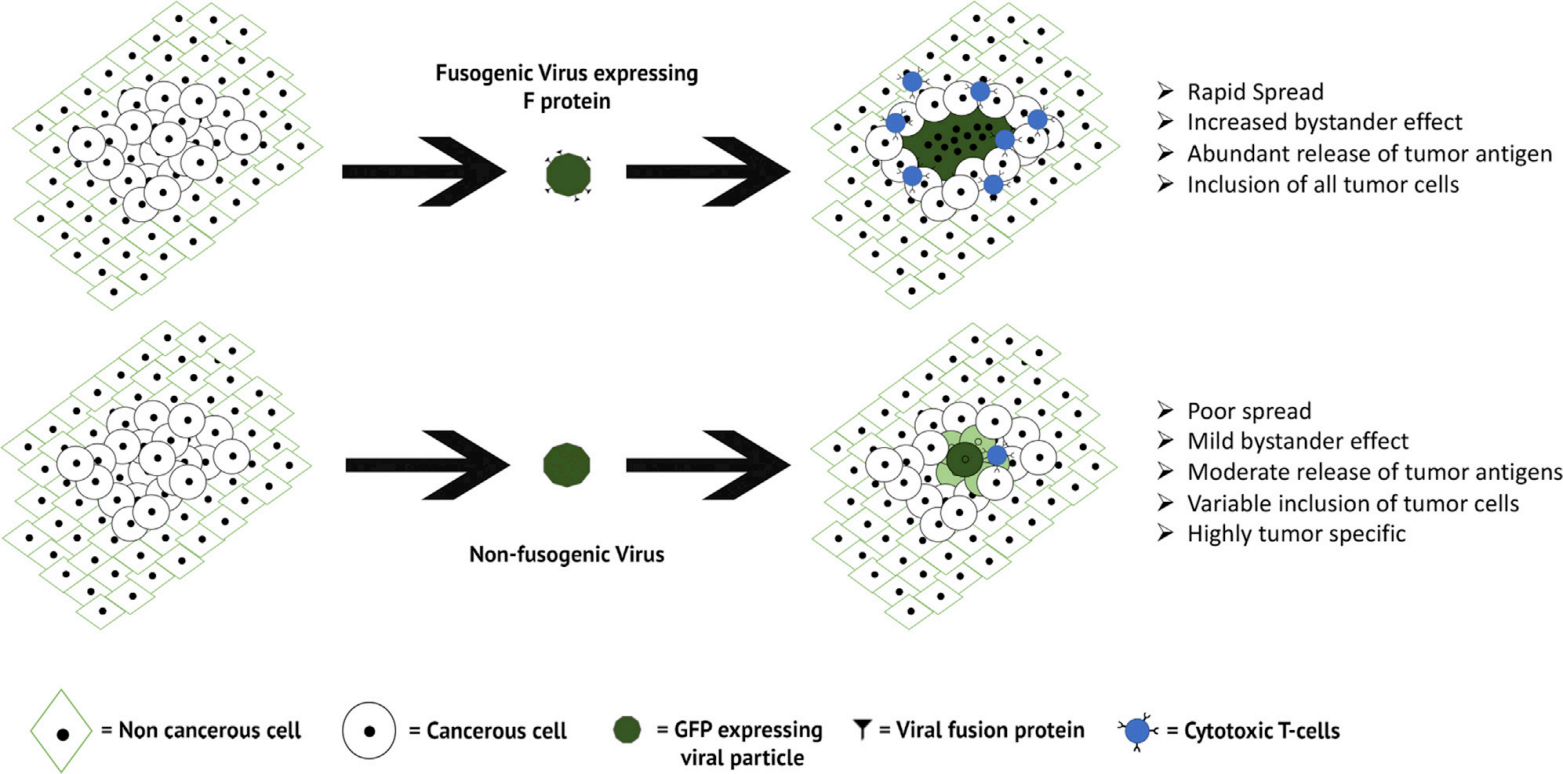
Schematic of Syncytia-Mediated Viral Spread During traditional viral infections, spread occurs slowly by repeated infections of single cells following production of new infectious progeny. In syncytia-mediated viral spread, dissemination is facilitated by the expression of the fusion protein on the infected cells, which in turn, binds to various receptors on the neighboring cells. By spreading through the interaction of the fusion protein, dissemination is both more rapid and no longer limited to cells that express the viral receptor. This results in infection of more tumor cells than with a nonfusogenic virus.

One phenotype, which has been suggested to improve oncolytic potential, is the ability of a virus to form syncytia. Syncytia are multinucleated cells created by the fusion of membranes from neighboring cells (Figure 1). Syncytia appear naturally during development, in the trophoblast, as well as in the development of the embryo.^8^^,^^9^ In other instances, however, such a phenotype results in either abnormal cell death or the spread of an infectious pathogen, such as a virus.^10^, ^11^, ^12^, ^13^ In 2002, a seminal paper by Fu and Zhang^14^ proposed that the induction of syncytia during OV might improve therapeutic efficacy both by enhancing viral spread within the tumor microenvironment as well as by inducing bystander killing of noninfected tumor cells. Since then, a number of naturally syncytia-forming viruses have been studied as potential oncolytic candidates.^15^ Additionally, a variety of nonsyncytia-forming viruses have been genetically engineered to induce artificially cell-cell fusion. Both types of viruses spread through *de novo* infection as well as fusion of infected and uninfected cells, which theoretically increases both their dissemination throughout the tumor and their overall efficacy. The purpose of this review is to discuss the capacity of different oncolytic viruses to form syncytia and how this ability influences each virus’s therapeutic potential.

### Natural Syncytia Viruses

Several viral families have evolved the ability to form syncytia between individual infected cells and neighboring uninfected cells. During infection, this phenomenon is facilitated by a viral fusion protein (often termed F) that mediates its function either with or without the presence of additional viral proteins.^16^^,^^17^ A number of these naturally occurring fusogenic viruses have been studied as oncolytic agents, including Newcastle disease virus (NDV), Sendai virus (SV), respiratory syncytial virus (RSV), and measles. In addition to these viruses, other viral families can induce membrane fusion between viral particles and cellular membranes without causing subsequent syncytia formation. For the purpose of this review, however, we will limit our discussion to viruses for which infection has been definitively shown to result in direct cell-cell fusion.

#### Newcastle Disease Virus

NDV represents one of the first oncolytic viruses to show clinical potential and has been studied for more than 60 years.^18^, ^19^, ^20^ NDV differentiated itself from other early oncolytic candidates both for its ability to infect a majority of human cancers, without the presence of a tumor-specific receptor,^21^ and for its lack of pathogenicity in humans.^22^ This allowed NDV to be used against a plethora of cancers with relative success.^23^, ^24^, ^25^, ^26^ From an oncolytic standpoint, at least part of the efficacy of NDV is achieved through virally induced apoptosis.^27^ Unfortunately, few studies have defined the mechanism through which NDV induces this pathway; however, it has been suggested that it is a cytotoxic effect of viral syncytial formation.^28^^,^^29^ It has been known for many years that NDV encodes a fusion complex that is normally involved in the fusion of the virion with a host cell during infection.^30^ For NDV, this fusion complex is made up of both the viral F (fusion) and HN (neuraminadase) proteins, with the F protein being initially transcribed as an inactive form (F_0_) and subsequently cleaved into the active polypeptides F_1_ and F_2_ by cellular proteases.^21^^,^^31^ Whereas the major evolutionary role of this fusion complex is most likely during viral entry, studies have also described high multiplicity NDV infections resulting in syncytia formation between infected and noninfected cells.^32^ Interestingly, during these infections, the relationship between F and HN plays a significant role in determining the outcomes of syncytia formation. The presence of both proteins results in efficient formation of stable syncytia, whereas mutations in one or both of these proteins can result in hyperfusogenic phenotypes that increase oncolytic potential.^33^, ^34^, ^35^ This suggests that the role of syncytia formation during oncolytic NDV treatment might vary based on the ratio of F to HN during a given infection. Some studies have attempted to bypass this issue by generating mutated forms of NDV that are hyperfusogenic due to an altered multibasic cleavage component of the F_0_ protein.^36^, ^37^, ^38^, ^39^ In these studies, the introduced mutations increased therapeutic efficacy by eliminating the need for the HN attachment protein. The above studies build off of an earlier study that shows that a mutation of lysine to arginine resulted in a mutated NDV that can mediate cell:cell fusion in the absence of HN.^40^ However it is important to mention that whereas this mutation allows for F to induce cell:cell fusion without HN, this occurs less efficiently than with HN present. This HN independence promoted NDV infection by decreasing the specificity of F protein binding, allowing for inclusion of a more heterogeneous tumor population. Surprisingly, these works also show that this increased fusion potential does not increase toxicity to healthy cells. These finding are significant since they provide evidence that virally induced fusion can be modified while still maintaining the cancer-specific tropism of oncolytic viruses in terms of the virus being able to replicate preferentially in tumor cells. Although this work establishes that oncotropic specificity can be maintained during syncytia formation, however, the molecular basis for this specificity was not determined.

In addition to the F:HN ratio, it has also been shown that the outcome of the fusogenicity of NDV depends on tumor cells’ resistance to apoptosis.^41^ Under normal conditions, NDV-induced syncytial formation rapidly activates apoptotic pathways, resulting in cellular death.^42^, ^43^, ^44^, ^45^ Although this is might seem an attractive outcome during OV, there is a fine balance in terms of cellular death and viral replication.^46^ In the case of fusogenic NDV, it is plausible that the increase in cellular death occurs too rapidly, thus preventing completion of the replication cycle. In contrast, expression of the anti-apoptotic protein B cell lymphoma-extra large (BCL-xL) during infection inhibits acute apoptotic cell death, which allows for more sustained viral replication.^41^^,^^47^ Over time, this decreased cell death actually results in improved viral spread and increased release of tumor antigens.^48^ These data suggest that the upregulation of BCL-xL might promote increased oncolytic potential of fusogenic NDV over time. Interestingly, it has previously been reported that the upregulation of BCL-xL is able to confer resistance to both multidrug and radiation therapy through a similar anti-apoptotic mechanism.^49^^,^^50^ By hijacking the tumors own cell-survival pathways, fusogenic NDV might therefore become an attractive treatment option for patients who have failed traditional therapies.

#### Sendai Virus

SV is a member of the same viral family as NDV, and the two viruses likely possess similar oncolytic characteristics.^51^ Compared to NDV, however, the oncolytic potential of SV is less well studied, although it also appears to have potential against a wide range of cancers.^52^^,^^53^ Like NDV, SV forms syncytia upon infection, which results in the induction of apoptotic cell death.^52^^,^^54^ Additionally, SV also encodes an accessory glycoprotein (G) HN, which aids in both virus:cell as well as cell:cell fusion,^55^, ^56^, ^57^ although it has been suggested that this protein is less important for the latter.^58^

Importantly, recent studies have directly addressed the role that syncytia formation plays in SV-based therapy.^53^^,^^54^^,^^59^ A publication by Hasegawa et al.^53^ demonstrates that the use of a fusogenic SV strain can increase both spread and cytotoxicity from viral infection by as much as four times over that of a nonfusogenic counterpart across a range of glioblastoma (GB) tumor lines. These results were then translated to an *in vivo* model using a virus also encoding an interferon (IFN)-β transgene, which resulted in significantly reduced tumor volume and improved overall survival compared to both mock and nonfusogenic controls.^53^ The authors of this work suggest that the efficacy of this fusogenic virus might be partially due to potential synergy between syncytia formation, which increased spread of the fusogenic virus, and the INF-β transgene, which enhanced overall anti-tumor immunity. In contrast, another possible explanation to why fusogenic SV shows increased efficacy was offered in a study by Suzuki et al.^60^ In this study, the research team found the presence of the SV-F protein itself resulted in an upregulation of interleukin-6 (IL-6) following treatment.^60^ Critically, IL-6 is associated with the inhibition of T-regulatory (Treg) cells, which are known to downregulate adaptive immune responses during immunotherapy. The fact that the F protein of SV can trigger IL-6 upregulation could therefore explain part of the increased efficacy seen with fusogenic SV. Whether this is an isolated phenomenon or whether all fusion proteins produce similar effects, however, remains to be determined.

#### Respiratory Syncytial Virus

RSV was originally identified as an oncolytic virus due to the degree of sensitivity that it exhibits toward IFN. As cancer cells have frequently lost the ability to respond to IFN, this allows for highly oncotropic infections using RSV.^61^ As with NDV and SV, the RSV-F protein is transcribed in an inactive form that then undergoes additional processing to the active form. In the case of RSV, F_0_ is cleaved by furin to produce the active complex consisting of two subunits: F_1_ a C-terminal membrane-anchored subunit, and F_2_, the N-terminal subunit. Like SV, RSV fusogenicity is not dependent on the presence of an HN protein, although this virus does encode a glycoprotein (G that can enhance its fusion capacity.^62^, ^63^, ^64^, ^65^, ^66^, ^67^, ^68^, ^69^ Theoretically, the ability of RSV to initiate fusion with neighboring cells, independent of coexpressed attachment proteins, could make this virus a more versatile inducer of syncytia formation; unfortunately, the direct role that syncytia formation plays in RSV-based oncolytics remains poorly defined

A 2015 paper by Choi et al.^70^ demonstrates that infection with RSV resulted in a reduction in the growth of some, but not all, hepatocellular carcinoma cell lines and that syncytia formation was specifically present in the cell lines that showed a decrease in cellular viability. A second study obtained similar results in a variety of skin carcinoma cell lines; however, this work also showed that infection with RSV resulted in increased apoptotic cell death that correlated with syncytia formation.^71^ Unfortunately, neither paper directly examines if there is a mechanistic relationship between syncytia formation and the inhibition of cancer cell growth; however, RSV-dependent syncytial formation has been shown to result in activation of p53, which could explain the apparent correlation.^72^^,^^73^

#### Measles Virus

The oncolytic potential of genetically modified measles virus (MV) has been known since the 1970s.^74^^,^^75^ Since then, the virus has been used against a wide range of cancers from many different tissue types, including lymphoma, leukemia, gliomas, and osteosarcoma.^76^, ^77^, ^78^, ^79^ MV is able to infect cells when its fusion complex interacts with one of three receptors on the target cell: CD46, signaling lymphocyte activation molecule (SLAM), or nectin-4.^80^, ^81^, ^82^, ^83^ MV then induces cell-to-cell fusion via expression of the MV-F protein and its interaction with the hemagglutinin protein H.^84^ Similar to other fusogenic viruses, MV-F is initially translated as an inactive form that cannot interact with the H protein to create the fusion complex unless MV-F is cleaved by the appropriate proteases during vesicular trafficking.^85^, ^86^, ^87^

A 2015 paper by Studebaker et al.^88^ shows the positive effect that fusogenicity has on oncolytic MV treatment of a typical teratoid rhabdoid tumor. Whereas this is a rare cancer subset, this work is interesting because it offers two key findings relevant to the use of syncytia forming oncolytic agents. First is that even a low MOI is sufficient to reduce tumor cell viability drastically after infection. Second is that the use of fusogenic virus *in vivo* can improve survival in both localized and metastatic tumor models. Similar results were also reported by the same group in the context of medulloblastoma.^89^ Together, these results show that the enhanced efficacy of fusogenic viruses is not strictly due to improvements in direct viral infection and spread alone but rather a combination of these mechanisms, along with the cytotoxic effects of noninfected cells, known as the bystander effect. Whereas the mechanism(s) mediating this bystander killing are likely multifactorial, efficacy does require viral replication and is associated with both increased cytotoxicity correlated with syncytial formation as well as activation of the Toll-like receptor 2 (TLR-2) antiviral signaling pathways.^79^ Critically, this work suggested that activation of TLR-2 signaling was caused by the mere presence of the H protein in the fusion complex.^90^, ^91^, ^92^ Similar to the induction of IL-6 by the SV-F protein, this result suggests that the presence of a fusion complex could itself be inflammatory, independent of either viral replication or cellular lysis. Interestingly, the authors of this work further suggest that in the case of MV, sensing of the F protein might be enhanced by the evolutionary interaction of the immune system with the human MV.^93^^,^^94^ Whether a similar effect is seen with the use of nonhuman oncolytic viruses is therefore a question that future studies should address.

Although both of the previous papers discuss how fusogenic MV can be used against fully differentiated tumor cell lines, tumor-initiating cells (TICs) pose a much greater challenge in terms of therapeutic outcomes and techniques.^95^, ^96^, ^97^ These cells are often resistant to most conventional treatments and are a primary cause of relapse for cancer patients.^6^^,^^98^^,^^99^ It is therefore interesting to note that MV-induced syncytia can apparently include gliomal TICs based on the inclusion of the CD133 marker.^78^ This work demonstrates a critical characteristic of syncytial-forming viruses in that they can often form syncytia with cells near them regardless of these cells’ susceptibility to direct viral infection. This allows syncytia-forming oncolytic viruses to spread to both differentiated tumor cells and TICs, offering a much greater therapeutic potential. Critically, the inclusion of normally noninfectible TICs in MV-induced syncytia appears at odds with the previously discussed retention of oncotropism following modification of virally induced fusion. Future studies are therefore needed to define the breadth and specificity of the fusogenic phenotype both *in vitro* and *in vivo*.

### Engineered Syncytia Viruses

With recent advances in molecular cloning and improved understanding of viral genomes, it is now possible to increase the oncolytic potential of many viruses through the addition or removal of specific genes. The goal of these changes is to increase or decrease phenotypes associated with strong oncolytic potential, including various aspects of the immune response (natural killer [NK] cell inhibition, CD8^+^ T cell activation, checkpoint blockade inhibition, etc.), lysis of infected cells, or spread within the tumor. One approach scientists have begun studying is to insert the fusogenic proteins of naturally occurring syncytia viruses into the proven backbones of nonfusogenic oncolytic viruses in order to enhance the spread of these viruses through the tumor. This combination allows for oncolytic viruses to benefit from the increased spread and lytic potential caused by syncytial formation while maintaining the inherent oncolytic properties of their nonfusogenic backbones. A variety of common vectors and fusion proteins have been used during these studies.

#### Recombinant Vesicular Stomatitis Virus

Vesicular stomatitis virus (VSV) is a cattle pathogen that is largely nonpathogenic in humans. Similar to RSV, VSV has been shown to replicate preferentially in tumor cells due to its restriction by functional IFN responses.^100^^,^^101^ Whereas VSV can cause membrane fusion between the virion and the host cell membranes, true syncytia formation is normally prevented by the presence of the fusion glycoprotein G, which is capable of initiating fusion only at acidic pH.^102^ In addition to its natural oncolytic potential, VSV is also frequently used to create recombinant viruses, since the virus offers significant freedom to add therapeutic genes to the genome without compromising other aspects of the viral biology.^103^

One example of such a recombinant virus is that of the VSV-H construct that encodes both the MV-F and H proteins into the VSV genome.^104^ This virus maintains the IFN-restricted replication properties of VSV,^105^ while adding the CD46-specific fusion mechanics of MV. Interestingly however, the new recombinant virus possesses greater all-around oncolytic capacity than either wild-type parental virus. This increased capacity is partially due to the replacement of the endogenous VSV-G protein with that of the MV fusion complex. With this replacement, VSV-induced membrane fusion is no longer limited by the pH dependence characteristic of the G fusion protein, giving the recombinant virus a more general fusion ability than either wild-type vector.^102^^,^^106^^,^^107^ This increased ability to produce syncytia results in the generation of significantly larger plaques than those seen with wild-type VSV, as well as syncytia formation that is independent of CD46 receptor density.^108^ A second recombinant VSV encoding only MV-F displayed a similar phenotype but also had improved cytotoxic effects against TICs, again independent of CD46-based viral entry, suggesting a second possible mechanism through which improved oncolytic potential might be obtained.^109^

A 2017 study by Le Boeuf et al.^110^ shows another recombinant VSV possessing a fusion-associated small transmembrane (FAST) protein. FAST proteins are F proteins that have been isolated from reovirus and are attractive from a genetic engineering perspective, since they are the smallest viral fusion proteins that allow for easy insertion into other oncolytic genomes.^111^ The results of this paper demonstrate that recombinant VSVs, which encode FAST proteins, are able to induce syncytial growth *in vitro*. The study then goes on to demonstrate that treatment with the recombinant virus leads to a reduction in the growth of established tumors *in vivo*. Additionally, VSV-FAST treatment also reduced the size and number of lesions in both metastatic breast and colon cancer models.^110^ This finding was not seen with nonfusogenic control viruses, suggesting that the ability of the recombinant virus to form syncytia may have induced an increased adaptive immune response, a hypothesis that was supported by VSV-FAST treatment, causing an increase in activated CD8^+^ and CD4^+^ T cells in both treated and nontreated tumors. Although this activation would start mainly in the treated tumors, as the adaptive immune response to the tumor escalates, these cells would localize into the metastatic lesions as well. This enhanced immune activation has been previously attributed to the rapid release of antigens that occurs during immunogenic death of the syncytia;^15^^,^^112^ however, our review suggests that other potential mechanisms, such as IL-6 or TLR activation, might also be involved.

Insertion of NDV-F has also been shown to improve the oncolytic potential of VSVs.^113^ In this work, mutated NDV-F was used, which allows for the induction of fusion, independent of the NDV-HN glycoprotein.^113^ Treatment with this virus resulted in both prolonged survival and increased viral infection in both metastatic liver and lung models compared to treatment with nonrecombinant controls. Importantly, as with the work on NDV, this study also demonstrates that altering virally fusogenicity does not compromise cancer tropism. In this case, the authors explicitly looked at the normal tissue surrounding the treated tumor to determine potential toxicities associated with introducing a fusogenic viral construct in an *in vivo* model; while robust infection of malignant tissue was observed, these studies demonstrated that infection was still excluded from surrounding normal cells. A later study using this same recombinant virus confirmed this result in metastatic colorectal cancer models.^114^

#### Recombinant Herpes Simplex Virus

Herpes simplex virus (HSV) is a member of the herpes virus family that is typically associated with dermal lesions (cold sores) in humans. Although certain strains of HSV can acutely form syncytia at physiological pH, the majority of HSV oncolytic trials, including the ones that led to the recent Food and Drug Administration (FDA) approval of Iymlygic,^115^ have been done using nonfusogenic recombinant HSV vectors armed with immune-modulating transgenes. In addition to the extensive body of oncolytic literature on nonfusogenic HSV, however, several studies have looked at the possibility of enhancing the normally low fusogenicity of HSV by adding fusogenic proteins into the HSV genome.

One such fusogenic virus is the recombinant HSV-GALV,^116^, ^117^, ^118^ which combines the oncolytic HSV backbone with the gibbon ape leukemia virus (GALV) fusion protein. Although GALV has poor oncolytic potential alone, the virus is hyperfusogenic, and insertion of its F protein into oncolytic HSV has been reported to generate a fusogenic construct with significantly increased oncolytic potential.^119^ Studies using this virus have shown that the addition of the GALV-F protein increases the death of infected tumor cells *in vitro* by up to 54%. Critically, as with work on fusogenic MV, these studies also observed substantial death of uninfected cells both *in vivo* and *in vitro* across several tumor models, including colorectal adenocarcinoma, GB astrocytoma, and lung epidermoid carcinoma. Since infection of 100% of malignant cells during oncolytic treatment has proven impossible to obtain, this enhanced bystander effect might represent a major mechanism through which fusogenic viruses achieved enhanced efficacy.

Fusogenic HSV was also used to study the potential impact of encoding two distinct fusion mechanisms into a single oncolytic virus.^120^ This work used a hyperfusogenic HSV backbone into which the authors additionally encoded the F glycoprotein of GALV.^14^ The proposed rationale for this approach was to combat the potential development of fusion resistance in treated tumors. Whether the development of this resistance is a problem during fusogenic OV remains unclear; however, the doubly fusogenic viruses did display enhanced efficacy in models of both breast and ovarian cancer. This improved efficacy correlated with increased cell death, release of virus into the tumor microenvironment at far greater levels (over 90% of the viral progeny), and higher rates of infection *in vivo*. Unfortunately, whereas the double fusion viral constructs resulted in overall impressive tumor control compared to untreated and nonfusion viral construct groups,^107^ no comparison to single fusogenic HSV constructs was included. Without this comparison, it is difficult to say how much added effect the addition of the second fusion protein has on the outcome of this model or if one such protein would have yielded the same results.

Finally, recombinant fusogenic HSVs have also been used to study the immunological ramifications of inducing syncytia formation.^61^^,^^121^^,^^122^ Similar to the work with VSV, these studies have generally shown that treatment with fusogenic HSVs induces more substantial and more effective anti-tumor immune responses than treatment with nonfusogenic controls. Critically, these results were found using HSVs encoding multiple distinct F proteins, which implies that the improved anti-tumor immunity is not specific to a single fusion protein, but instead represents an inherent benefit of syncytial induction. No mechanism for this enhanced immune activity has been discovered to date; however, one proposal is that it is related to fusogenic viruses improved with bystander killing. In this model, more cells being killed results in more antigens from the tumor able to be presented on antigen-presenting cells. This results in improved adaptive immunity, particularly against subpopulations of the tumor that were previously uninfectable by the virus. Alternatively, it has also been proposed that the formation of unique antigen-rich vesicles might play a role.^61^^,^^123^ These vesicles, known as syncytiosomes, are released at an increased rate from syncytial bodies and are then uptaken by antigen-presenting cells, resulting in increased cross presentation of tumor-associated antigens.^61^ Unfortunately, neither of these hypotheses has yet to be definitively proven in the context of OV. Therefore, whereas multiple studies point to syncytial formation inducing improved anti-tumor immunity, this phenomenon remains poorly understood and should be a focus of future studies.^111^^,^^124^

#### Recombinant Adenovirus

Adenovirus (Ad) is a member of the *Adenoviridae* family, which is characterized as nonenveloped, double-stranded DNA viruses. Oncolytic Ad has shown promising results across a variety of tumor models with many of the studies focusing on a common human variant, Ad subtype 5 (Ad5).^125^ Although Ad5 is normally, completely nonfusogenic, it can be made to induce syncytia by the addition of exogenous F proteins. In the case of Ad5, many of these studies have encoded various fusion proteins into replication-defective variants of the virus as a form of gene therapy. As this is not a true form of oncolytics, we have excluded these works form our current review; however, it should be noted that many of these papers show increased efficacy using fusogenic, nonreplicating Ad vectors, which supports the conclusion that formation of syncytia alone can be a potent mediator of anti-tumor efficacy.

The earliest study combining replication-competent Ad and syncytia formation used the unique approach of oncolytic Ad5 together with plasmid DNA encoding the GALV-F protein to induce syncytia formation. Similar to results with actual recombinant fusogenic viruses, this study found the exogenous addition of F protein increased both viral spread and cytotoxicity *in vitro* and impressively, translated into complete cures of both small and large established tumors in an *in vivo* model.^126^ Later studies confirmed that this efficacy could be duplicated in multiple cell lines when the GALV-F protein was inserted directly into the Ad genome,^119^^,^^127^^,^^128^ while still maintaining oncolytic safety. Interestingly, in contrast to previous studies using GALV-F, the efficacy of this fusogenic Ad appears to be strictly due to improved viral spread, since the immunological responses induced following treatment did not differ with the addition of the F protein.

### Conclusions

Syncytia are defined as the fusion of multiple cells into a single multinucleated cell body. From an evolutionary perspective, the likely intention of virally induced syncytia formation is to increase the spread of a virus. Thus, the induction of this process represents a novel solution to one of the biggest challenges facing OV. By utilizing fusogenic viruses for OV, scientists have proven that forcing syncytia formation both can and will kill tumor cells regardless of tumor type. Although these results show the promise of syncytial-forming viruses, many questions remain about the mechanisms involved following therapy. In particular, the mechanisms mediating tumor specificity during fusogenic infection and the ability of these viruses to induce bystander killing and enhanced anti-tumor immunity remain to be determined. Additionally, although a variety of fusion proteins have been shown to enhance nonfusogenic oncolytic viruses, whether any of these has an inherent advantage compared to the others remains unclear. Therefore, whereas multiple studies have conclusively shown the promise of inducing syncytia formation during OV, additional mechanistic work is likely needed to maximize this strategy in the future.

## Author Contributions

C.B. researched and prepared manuscript. E.B. oversaw the project and prepared the manuscript.

## Conflicts of interest

The authors declare no competing interests.
